# Social Tolerance in Wild Female Crested Macaques (*Macaca nigra*) in Tangkoko-Batuangus Nature Reserve, Sulawesi, Indonesia

**DOI:** 10.1002/ajp.22114

**Published:** 2013-01-10

**Authors:** Julie Duboscq, Jérôme Micheletta, Muhammad Agil, Keith Hodges, Bernard Thierry, Antje Engelhardt

**Affiliations:** 1 Junior Research Group for Primate Sexual Selection, Reproductive Biology Unit, German Primate CenterGöttingen, Germany; 2 Courant Research Centre for the Evolution of Social Behaviour, Georg-August UniversityGöttingen, Germany; 3 Department Ecology, Physiology and Ethology, IPHC, Centre National de la Recherche Scientifique, Université de StrasbourgStrasbourg, France; 4 Department of Psychology, Centre for Comparative and Evolutionary Psychology, University of PortsmouthUnited Kingdom; 5 Reproductive Biology Unit, German Primate CenterGöttingen, Germany; 6 Faculty of Veterinary Medicine, Bogor Agricultural UniversityBogor, Indonesia

**Keywords:** females, social behavior, social tolerance, comparative studies, macaques, *Macaca nigra*

## Abstract

In primates, females typically drive the evolution of the social system and present a wide diversity of social structures. To understand this diversity, it is necessary to document the consistency and/or flexibility of female social structures across and within species, contexts, and environments. Macaques (*Macaca* sp.) are an ideal taxon for such comparative study, showing both consistency and variation in their social relations. Their social styles, constituting robust sets of social traits, can be classified in four grades, from despotic to tolerant. However, tolerant species are still understudied, especially in the wild. To foster our understanding of tolerant societies and to assess the validity of the concept of social style, we studied female crested macaques, *Macaca nigra*, under entirely natural conditions. We assessed their degree of social tolerance by analyzing the frequency, intensity, and distribution of agonistic and affiliative behaviors, their dominance gradient, their bared-teeth display, and their level of conciliatory tendency. We also analyzed previously undocumented behavioral patterns in grade 4 macaques: reaction upon approach and distribution of affiliative behavior across partners. We compared the observed patterns to data from other populations of grade 4 macaques and from species of other grades. Overall, female crested macaques expressed a tolerant social style, with low intensity, frequently bidirectional, and reconciled conflicts. Dominance asymmetry was moderate, associated with an affiliative bared-teeth display. Females greatly tolerated one another in close proximity. The observed patterns matched the profile of other tolerant macaques and were outside the range of patterns of more despotic species. This study is the first comprehensive analysis of females’ social behavior in a tolerant macaque species under natural conditions and as such, contributes to a better understanding of macaque societies. It also highlights the relevance of the social style concept in the assessment of the degree of tolerance/despotism in social systems. Am. J. Primatol. 75:361-375, 2013. © 2013 Wiley Periodicals, Inc.

## INTRODUCTION

In nonhuman primates, females typically drive the evolution of the social system, highlighting the importance of focusing on females when studying primate social evolution [Clutton-Brock & Lukas, 2012; Lindenfors et al., 2004]. Female primate social structures vary greatly between species, ranging from females forming loose and changing associations, to females establishing stable bonds with a subset of partners [Wrangham, 1980]. Different conceptual frameworks exist to explain the evolution of this social diversity. Variation in social structures may reflect ecological pressures—mainly predation and food abundance and distribution—which would shape not only the grouping patterns of females (i.e., dispersal vs. philopatry) but also their social relationships [Koenig, 2002; Sterck et al., 1997; van Schaik, 1989]. Alternatively or additionally, relations between behavioral traits, phylogenetic constraints and/or self-organizing principles may limit the flexibility and plasticity of social structures, and thus constrain their evolution [Hemelrijk, 1999; Thierry, 2007]. Quantifying the consistency and/or flexibility of female social relationships across contexts and environments, within and across species, is a necessary strategy in understanding their evolution.

Female primate social structures result from a complex combination of cooperative and competitive interactions [Hinde, 1976]. Furthermore, an individual's social behavior is influenced both by ecological and social pressures, that is, the strategies of conspecifics [Wrangham, 1987]. Thus, previous studies on female-bonded groups (i.e., with female philopatry) have not only investigated ecological factors influencing female social relationships [Koenig, 2002], they also have looked at the various social trade-offs faced by females in terms of cooperation and competition. One such trade-off is exemplified by the degree of social tolerance between dominant and subordinate individuals, which has shaped alternative conflict management strategies [de Waal, 1986], tightly linked to sociality, and potentially, differential access to resources, whether social or ecological [van Schaik, 1989].

Macaques (*Macaca* sp.) are an ideal taxon in which to investigate the determinants of social behavior because they show both consistency and variation in their social relations. They also live in a great variety of environments [Fooden, 1982]. Most macaques form multimale multifemale groups. Males emigrate upon reaching sexual maturity, whereas philopatric females organize themselves into matrilines, that is, subgroups of maternal kin [Pusey & Packer, 1987]. However, patterns of aggressive, submissive, and affiliative behaviors, the degree of intensity and symmetry in social interactions, and conciliatory tendencies vary considerably among species [Thierry, 2007]. Distinctive social styles, that is, sets of interrelated social traits, are discernible and categorized into four social grades, ranging from despotic to more tolerant [de Waal, 1989; Thierry, 2000; Thierry, 2007]. Despotic species such as rhesus and Japanese macaques (*Macaca mulatta* and *M. fuscata*) engage in conflicts of high intensity, mainly unidirectional, and seldom reconciled [Chaffin et al., 1995; de Waal & Luttrell, 1989]. There is pronounced dominance asymmetry between individuals and the silent bared-teeth display serves as a formal submissive signal [de Waal & Luttrell, 1985; Preuschoft & van Schaik, 2000]. In these species, dominance rank and kinship markedly constrain social interactions [Chapais, 1983]. In contrast, more tolerant species such as Tonkean and moor macaques (*M. tonkeana* and *M. maurus*) display less severe, more bidirectional, and more frequently reconciled conflicts [Demaria & Thierry, 2001; Petit et al., 1997; Thierry, 1985]. Dominance asymmetry is less pronounced, and the silent bared-teeth display is mainly used in affiliative interactions [Petit & Thierry, 1992; Thierry et al., 2000a]. Affiliation occurs in an extended network of partners with limited influence of dominance and kinship relationships [Matsumura and Okamoto, 1997; Thierry et al., 1994].

The concept of an evolved grade-specific social style implies that within a species, interrelated social traits are robust despite variation in the environment. It also implies that social variation within a grade is less pronounced than between grades. So far, most of the knowledge accumulated on macaque societies has come from studies conducted either in captive or provisioned settings, where behaviors and/or the dynamic of interactions may be altered [Asquith, 1989; Judge, 2000]. Furthermore, most studies to date have focused on the despotic end of the social style spectrum (grades 1 and 2) whereas the most tolerant one, grade 4, is the least studied grade [Thierry, 2007]. Research in captive settings has shown that the behavioral profile of tolerant macaques differs substantially from that of the more despotic rhesus and Japanese macaques for example [Petit et al., 1997; Thierry, 1985]. Information on wild tolerant macaques is however limited to few studies on the same group of provisioned moor macaques, which showed differences relative to captive populations. Counter aggression, for instance, was, unexpectedly, not observed [Matsumura, 1996, 1998; Matsumura & Okamoto, 1997]. Whether counter aggression is really absent in this species or whether this finding is related to difficult observation conditions or to the inadequacy of the data set is unknown. More information on tolerant macaques under natural conditions in the wild is clearly needed.

To extend our knowledge of social behaviors of tolerant macaque species in the wild, we studied female social behaviors in two wild, habituated but not provisioned groups of crested macaque, *M. nigra* [Fooden, 1969], a member of grade 4, in Sulawesi, Indonesia. We first investigated female aggressive and affiliative behavioral patterns to assess social tolerance focusing on the frequency, intensity, and distribution of agonistic and affiliative behavior among females, their dominance gradient, and their level of conciliatory tendency. We studied the context and directionality of the silent bared-teeth display to verify that it constitutes an affiliative rather than a submissive signal in this species. We also analyzed further behavioral patterns that have not yet been assessed in grade 4 macaques, although researchers have used them to characterize social tolerance in other grades: responses to others’ approach, and distribution of affiliative behavior across partners. We then examined whether the patterns observed in the wild population were consistent with those reported in captivity. Finally, to evaluate our results in the framework of macaque social styles, we compared them to data from other grade 4 macaques and from other species of other grades. We expected the wild population's behavioral profile to be consistent with captive conspecifics and other grade 4 macaque populations but substantially different from macaques of other grades.

## METHODS

### Study Site and Groups

Crested macaques are endemic to the island of Sulawesi, Indonesia [Sugardjito et al., 1989]. The study population inhabits the Tangkoko-Batuangus Nature Reserve (1°33′N, 125°10′E; e.g., [Neumann et al., 2010]), broadly classified as a lowland rainforest with seasonal variation in rainfall and fruit abundance [O'Brien & Kinnaird, 1997]. The research area is a mix of primary and secondary forest as well as old regenerating gardens [O'Brien & Kinnaird, 1997]. The study was part of the Macaca Nigra Project (http://www.macaca-nigra.org), a long-term field project focusing on the biology of crested macaques.

We studied two groups, “PB” and “R1,” comprising about 60 and 80 individuals, respectively. The monkeys were well habituated to human observers, but not provisioned, and spent around 60% of their time on the ground [O'Brien & Kinnaird, 1997]. We could individually identify all adults based on physical characteristics (shape and color of the anogenital region, wrinkles and special facial features, or scars).

### Data Collection

JD, JM, and another field assistant followed each study group from dawn (*ca*. 5:30 am) to dusk (*ca*. 6:00 pm) every day between October 2008 and May 2010. We collected behavioral data on all adult females (15–18 in PB, 21–24 in R1) using focal animal sampling [Martin & Bateson, 1993] (interobserver reliability: Cohen's kappa = 0.69–0.90, correlation coefficients between behavioral variables = 0.79–0.98, all *P* < 0.05). Each day, we selected females for observation in a predetermined random order, balancing observations across four periods of the day (early and late morning and early and late afternoon). For each subject on a given day, we aimed at collecting 30 consecutive point samples for her activity. Sometimes, we could not accurately monitor the focal female's activity (e.g., she was temporarily out of sight). In such cases, we extended the observation protocol as long as necessary to achieve 30 data points of activity monitoring. We also extended protocols to get postconflict (PC) observation periods of sufficient length (see section Conciliatory tendency). We included all focal protocols lasting more than 2 min in the analyses. Focal follows in the final data set had the subject in sight (whether her activity was visible or not) for a median of 32 min (range: 2–100 min, including one outlier that lasted almost 2 hr when we monitored a female about to give birth); these records included a median of 30 activity point samples (range: 2–84). We recorded the subject's activity (feeding, foraging, socializing, traveling, resting) every minute and the identity of neighbors (in body contact, within one body length, and within five body lengths) every alternate minute. We recorded focal social events continuously, including start and end time of the interactions, the sequence of all of the subject's behaviors (see next section), as well as identity and behaviors of all social partners. In addition, every 10 min, we recorded the identity and general activity of neighbors up to ten body lengths away, and every 30 min, we noted the general activity of the majority of visible individuals around the focal female (usually up to 20 m).

During the study, several females reached adulthood (gave birth to a live infant for the first time) and one disappeared. For greater clarity, we excluded these females from our analysis. Thus, in total, our study included 2,480 hr of focal data from 36 subjects (PB: median = 68 hr per female (range: 65 – 78, *N* = 15); R1: median = 66 hr per female (range: 59 – 71, *N* = 21)).

### Behavioral Definitions

We defined an *aggressive interaction* as the display of an aggressive behavior of any intensity followed by an aggressive or nonaggressive response. Aggressive acts ignored by the receiver contributed only to our analysis of responses to aggression (see below). Aggressive behaviors included *threat*: aggressive vocalizations (bark, grunt, rattle, scream) and/or facial expressions (half-open mouth, open-mouth bared-teeth, stare, jaw movement); *attack*: aggressive behaviors exceeding the threat intensity but excluding bite, further divided into *contact attacks* (hit, missed hit, grab and push) and *noncontact attacks* (chase, lunge, and stamp); and *bite* [Thierry et al., 2000a].

We defined a *displacement*, or *approach-retreat interaction*, as a female approaching without provocation, usually within five body lengths, another female who simultaneously moved away [Thierry et al., 2000a]. Displacements did not involve any aggressive component.

*Affiliative interactions* included grooming, nonaggressive body contact, embrace, tail grasp/rub, soft grunt, and affiliative facial expressions such as lipsmack [Thierry et al., 2000a]. We counted as one grooming bout any continuous episode of grooming, by one or both partners, with breaks not exceeding 10 sec.

We also recorded *approach* to one body length of another female, as long as the two stayed within this distance for at least 5 sec. Only *nonagonistic approaches*, where the approaching female did not direct aggression to her partner while coming near, were used when evaluating the response of an approached individual [de Waal & Luttrell, 1989].

Finally, we recorded *silent bared-teeth display*, a facial expression where the upper lip or both lips are vertically retracted, exposing the teeth and sometimes the gums [Thierry et al., 2000a].

This research adheres to the legal requirements of the German and Indonesian governments, and adheres to the American Society of Primatologists Principles for the Ethical Treatment of Non-Human Primates.

### Data Analysis

Analyses were limited to dyadic interactions involving focal females. When more than two individuals were involved sequentially or simultaneously with the focal female, we broke down polyadic interactions into dyadic ones or we prioritized interactions according to the intensity of behaviors used: agonistic interactions were prioritized over affiliative ones and interactions involving body contact were prioritized over those involving only displays. We calculated frequencies (per hour of observation time, that is, total number of minutes across all focal protocols, divided by 60, the focal female was visible, with or without the possibility of monitoring her activity) and percentages (of behavior as a proportion of interactions) per focal female, and then computed medians and ranges as well as means ± SDs across all females in each group.

#### Agonism

To assess the intensity of aggression, we calculated median percentages of specific aggressive behaviors (threat, noncontact attack, contact attack, and bite) as a proportion of all aggressive interactions for each female. If an interaction included several aggressive elements, we categorized it by the most intense aggression shown (bite > contact attack > noncontact attack > threat). To analyze response to aggression, we categorized the responses as *leave* (move away from the aggressor from any proximity category to a larger distance), *retaliate* (respond aggressively to the aggressor), *affiliate* (respond with a friendly behavior), or *ignore (*no reaction or change in activity). We quantified counter-aggression as the proportion of aggressive interactions with counter aggression (any aggressive response, including aggressive screams).

#### Affiliation and other behaviors

To assess the intensity of affiliation, we calculated the median percentage of affiliative interactions with body contact (e.g., touch, embrace, tail grasp/rub, grooming) as a proportion of all affiliative interactions for each female. If a given interaction included several affiliative elements, we prioritized body contact over other behavior.

To assess the degree of tolerance among females, we grouped responses to nonagonistic approach as having a *negative outcome* if the approached female retreated, aggressed, or screamed at the approaching female, a *positive outcome* if the two females engaged in affiliation, and a *neutral outcome* if there was no action/reaction from either female [Cooper & Bernstein, 2008; de Waal & Luttrell, 1989]. To measure how evenly females distributed their grooming bouts and approaches among adult female group-mates, we used the standardized Shannon–Wiener diversity index *H*/*H*_max_ [Cheney, 1992; Shannon & Weaver, 1949]. This index is calculated as follows:


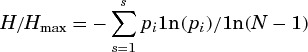


where *s* is the number of actual interaction partners, *p*_i_ the relative proportion of behavior exchanged (i.e., proportion of total grooming bouts, or proportion of total approaches) between the *i*th focal female and other females, and *N* the total number of potential female partners, that is, the number of females in the group. This index ranges from 0 (very uneven distribution of the behavior) to 1 (even distribution across female group-mates).

#### Conciliatory tendency

PC observations were extracted from focal protocols and did not differ from a normal protocol in terms of data collected. PCs started right after the last exchange of aggressive behaviors between the focal female and her opponent and lasted ideally 10 min (range: 2–10 min). Usually, matched-control observations (MC) are conducted at the same time the next possible observation day after the aggressive interactions [de Waal & Yoshihara, 1983]. Because this procedure did not guarantee ideal matching conditions, we chose MCs a posteriori [Aureli, 1992] from focal protocols conducted within a month before or after the protocol in which the particular aggressive interaction was recorded (median number of days between PC and MC: 17.4 (range: 1–32)). To qualify as MC, the same two opponents as in the PC had to be in proximity (<10 body lengths), and the group's general activity had to be the same in the MC as in the PC. In addition, neither of the two opponents should have been involved in aggressive interactions within 2 min prior to or after the beginning of the MC, nor should they be engaged in affiliation with each other. We compared the occurrence of the first affiliation between opponents between PC and MC periods: we classified pairs as “attracted” when the first affiliation occurred sooner in the PC compared to the MC, “dispersed” when the first affiliation occurred sooner in the MC compared to the PC or “neutral” when the first affiliation occurred at the same time in both periods or no affiliation occurred in either period. We computed the corrected conciliatory tendency (CCT) as the number of attracted minus dispersed pairs divided by the total number of pairs [Veenema et al., 1994], first per individual, then across females (median). The CCT was calculated separately for contact affiliations only and for all affiliations together to ensure valid comparisons with other studies.

#### Dominance hierarchy

Interaction matrices used for calculating hierarchy parameters were based on two types of dyadic interactions extracted from focal protocols: *displacements* and *winner–loser interactions*; the latter were “decided” aggressive interactions with a clear outcome, mainly interactions in which one of the opponents left (this species has no obvious submissive signals; [Thierry et al., 1994; Thierry et al., 2000a]). If other responses occurred, such as affiliation or retaliation, we coded the interaction as undecided. Displacements were always decided interactions.

We assessed hierarchy linearity with the *linearity index* h′ (corrected for unknown relationships, [de Vries, 1995]), which ranges between 0 (not linear) and 1 (strictly linear). To assess power asymmetries among females, we calculated the *Directional Consistency Index (DCI)*, which represents how often a particular behavior is given in the most frequent direction and ranges from 0 (equal exchange) to 1 (unidirectional) [van Hooff & van Wensing, 1987]. We also calculated *steepness*, based on normalized David's scores [Gammell et al., 2003], which measures the degree to which individuals differ in their ability to win contests [de Vries et al., 2006]: steepness can vary from 0 (no average difference) to 1 (maximum average difference). We report steepness calculations based on both *D*_ij_ indices, which take into account the frequency of interactions, and *P*_ij_ indices, which do not [de Vries et al., 2006]. The displacement matrices served as the basis for calculating female ranks according to the I&SI method (Matman 1.1 [de Vries, 1998; de Vries et al., 1993]).

As 54% of aggressive interactions were “undecided,” we also derived a hierarchy (I&SI method) and calculated DCI and steepness based on *all initiated aggressive acts*, that is, all aggressive acts given, regardless of the response of the receiver but excluding ignored aggressive acts. We compared the two indices and the rank order obtained to those based on displacement and winner–loser interactions to explore the effect of undecided interactions on power asymmetries and to assess the reliability of displacement interactions in building hierarchies in this species [de Waal, 1989; Thierry et al., 1994]. We also give information on some descriptive attributes of the matrices (Appendix Tables AI–AVI), such as number of interactions recorded, percentage of unknown relationships (dyads with no interaction), and percentage of two-way relationships (each dyad member both won and lost contests against her opponent).

#### Silent bared-teeth display

To analyze the context in which silent bared-teeth displays occurred, we categorized the occurrences of this facial expression into three mutually exclusive contexts, according to how the two females interacted 10 sec before and/or after the display: *negative* (aggression), *positive* (affiliation), or *neutral context* (approach or sit in proximity, without further social exchange). We assessed the distribution of the bared-teeth display across partners with the standardized Shannon–Wiener diversity index (see above). To test the directionality of the display relative to females’ dominance rank, we calculated the *up/down index*, which measures how consistently a behavior is directed up or down the hierarchy [Castles et al., 1996; de Waal & Luttrell, 1989]. The up/down index was computed as follows for each individual: 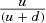, where *u* is the proportion of displays given up the hierarchy and *d* the proportion given down the hierarchy. An index of 0.5 indicates no bias and constitutes the reference point; an index lower than 0.5 indicates a tendency to express the behavior down the hierarchy, and vice versa [Castles et al., 1996].

### Comparative Perspective within the Macaque Genus

We evaluated our results in the context of variation among macaque societies by contrasting them with data on a specific set of behavioral variables from other macaque populations. We first assessed the degree of tolerance of the study females relative to other grade 4 macaque species. In this comparative data set, a single research team conducted all but one study (on moor macaques) ensuring comparable definitions and data collection methods [Petit et al., 1997; Petit & Thierry, 1994; Thierry, 1985; Thierry et al., 1994]. For the comparison with macaque species of other grades, we mainly selected studies conducted under natural conditions, but also included those in which provisioning occurred to increase our sample size. We included studies that matched behavioral definitions to ours as much as possible, and included focal female data only.

### Statistical Analyses

We based statistical analyses on individual data for each group separately. We tested for group differences in rates and percentages of behaviors and interactions. When groups did not differ statistically, we combined the results for subjects in both groups to simplify presentation, but still report the group-wise statistics. When groups showed significant differences, we present the results separately for each group. For indices of distribution (Shannon–Wiener and up/down indices), indices of dominance gradient (DCI and steepness) and conciliatory tendency, we always report results for each group separately.

We ran most analyses in R 2.14.2 [R Development Core Team 2011]. All tests were nonparametric, exact (package “exactRankTests” [Hothorn & Hornik, 2011]), and two tailed. We analyzed differences across outcomes of approaches and across contexts of silent bared-teeth occurrences based on frequencies of each outcome or context per observation time. More specifically, we tested whether each outcome of an approach was equally likely and whether females showed silent bared-teeth in each context equally. When the difference between the three outcomes or contexts was significant, we ran post-hoc tests with the function friedmanmc (package “pgirmess” [Giraudoux, 2012]) to determine which outcome of approaches was more likely or which context the silent bared-teeth was more likely associated with. We give the observed difference and the critical difference. When the former is higher than the latter, the difference between the two categories compared is considered significant [Siegel & Castellan, 1988]. We used Matman 1.1 with 2,000 permutations to calculate the linearity index h′, DCI and percentages of unknown and two-ways relationships (Noldus, Wagenigen, [de Vries et al., 1993]). Steepness was calculated and tested in R (package “steepness” [Leiva & de Vries, 2011]) with 2,000 permutations. All significance levels were set to 0.05.

## RESULTS

### Agonism

**Fig. 1:**
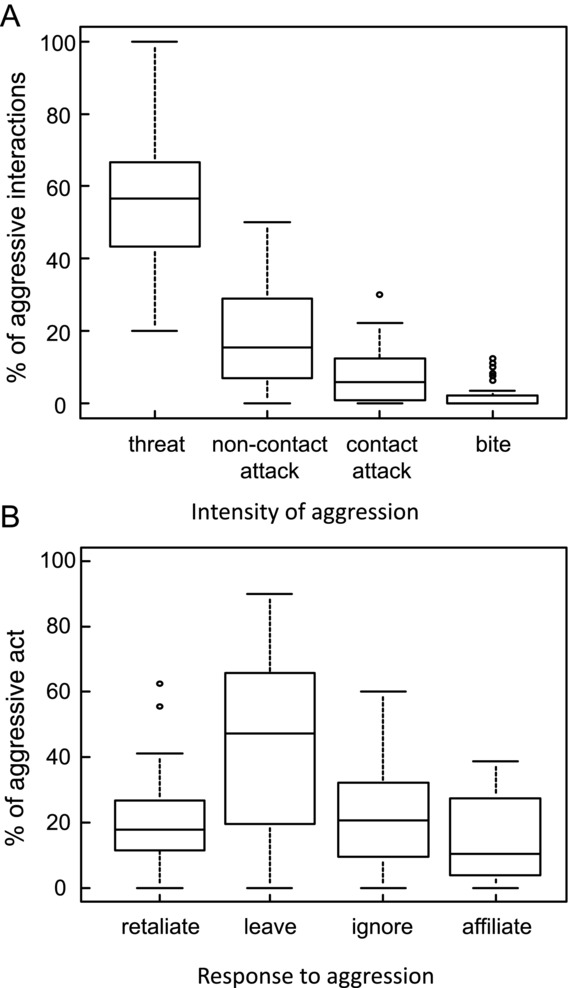
Detailed characteristics of agonistic interactions (see definition in text): intensity of aggression as a proportion of all agonistic interactions (A) and response to aggression as a proportion of all aggressive acts (B) across the two groups combined (median, interquartiles and 1.5 interquartile range, *N* = 36 females).

Females were involved in an aggressive interaction with another female about once every 3 hr (Table I). Displacements and aggressive interactions occurred at similar rates (Table I). Most aggressive interactions involved only threats (Table I, Fig. 1A). Noncontact attacks occurred more frequently than contact ones (Table I, Fig. 1A). Females rarely bit each other (Table I, Fig. 1A).

**Table I:** Summary of Behavioral Data in the Two Study Groups

	Groups			
	
	PB		R1	
		
	Median (range)	Mean ± SD	Median (range)	Mean ± SD
Agonism				
Agonistic interaction (nb/hr)	0.26 (0.18–0.58)	0.31 ± 0.12	0.38 (0.20–0.95)	0.43 ± 0.19
Displacement (nb/hr)	0.28 (0–1.22)	0.34 ± 0.33	0.48 (0.27–1.09)	0.50 ± 0.21
Aggressive behavior (percentage of all agonistic interactions)				
Threat	67 (44–91)	68 ± 16	61 (20–89)	61 ± 19
Noncontact attack	20 (0–44)	18 ± 13	25 (5–50)	27 ± 14
Contact attack	11 (0–50)	12 ± 13	8 (0–30)	10 ± 9
Bite	0 (0–9)	2 ± 3	0 (0–13)	3 ± 5
Response to aggression (percentage of all aggressive acts)				
Avoid	36 (0–87)	24 ± 17	56 (0–90)	46 ± 27
Retaliate	20 (4–36)	20 ± 9	14 (0–63)	21 ± 17
Affiliate	11 (0–38)	15 ± 14	10 (0–39)	14 ± 13
Ignore	21 (0–60)	24 ± 17	20 (0–63)	19 ± 14
Counter aggression	30 (4 – 50)	28 ± 15	21 (0 – 71)	26 ± 19
Affiliation and other				
Affiliative interaction (nb/hr)	2.19 (1.30–3.33)	2.25 ± 0.60	2.95 (0.98–4.24)	2.73 ± 0.76
Contact affiliation (percentage of all affiliations)	63 (20–56)	64 ± 6	63 (48–72)	62 ± 7
Grooming bout (nb/hr)	1.25 (0.65–1.89)	1.10 ± 0.55	1.22 (0.22–2.50)	1.25 ± 0.63
Grooming H′/H_max_	0.86 (0.77–0.94)	0.86 ± 0.05	0.85 (0.67–0.92)	0.84 ± 0.07
Approach (nb/hr)	4.95 (3.11–7.90)	5.28 ± 0.35	5.00 (2.48–8.88)	5.67 ± 0.43
Approach H′/H_max_	0.94 (0.93–0.97)	0.95 ± 0.01	0.92 (0.87–0.97)	0.92 ± 0.02
Outcome of approach (percentage of all nonagonistic approaches)				
Negative	7 (1–24)	9 ± 3	13 (6–23)	13 ± 3
Positive	28 (21–42)	30 ± 5	29 (21–40)	30 ± 3
Neutral	63 (49–71)	62 ± 6	58 (44–66)	57 ± 4
Silent bared-teeth (nb/hr)	0.15 (0.05–0.49)	0.17 ± 0.02	0.12 (0.05–0.32)	0.16 ± 0.02
Silent bared-teeth H′/H_max_	0.63 (0.24–0.83)	0.68 ± 0.03	0.52 (0–0.79)	0.49 ± 0.23
Context of occurrence of silent bared-teeth (percentage of all occurrences)				
Negative	9 (0–40)	13 ± 14	13 (0–53)	15 ± 16
Positive	45 (20–87)	49 ± 19	59 (25–100)	57 ± 23
Neutral	36 (9–80)	36 ± 19	29 (0–63)	28 ± 19
Silent bared-teeth up/down index	0.50 (0.14–1)	0.56 ± 0.28	0.50 (0–1)	0.49 ± 0.36

Hourly frequencies (nb/hr), percentages as proportion of specific interactions/behaviors considered, Shannon–Wiener Diversity Index H′/H_max_ and up/down index (median (range) and mean ± SD, *N*_PB_ = 15, *N*_R1_ = 21).

Recipients of aggression usually responded by leaving the aggressor's proximity (Table I, Fig. 1B). Less frequently, they retaliated, ignored their aggressor, or attempted to appease her with friendly behavior (Table I, Fig. 1B). Females counter-attacked their opponents in 27% of aggressive interactions (median; range: 0–71%, mean = 27% ± 17 SD, *N* = 36; Table I).

### Affiliation and Approach

Females had affiliative interactions 2.5 times per hour and more than 60% of these interactions involved body contact (including grooming; Table I). Females were involved in a grooming bout with another female about once an hour and they closely approached female partners approximately five times per hour (Table I).

**Fig. 2:**
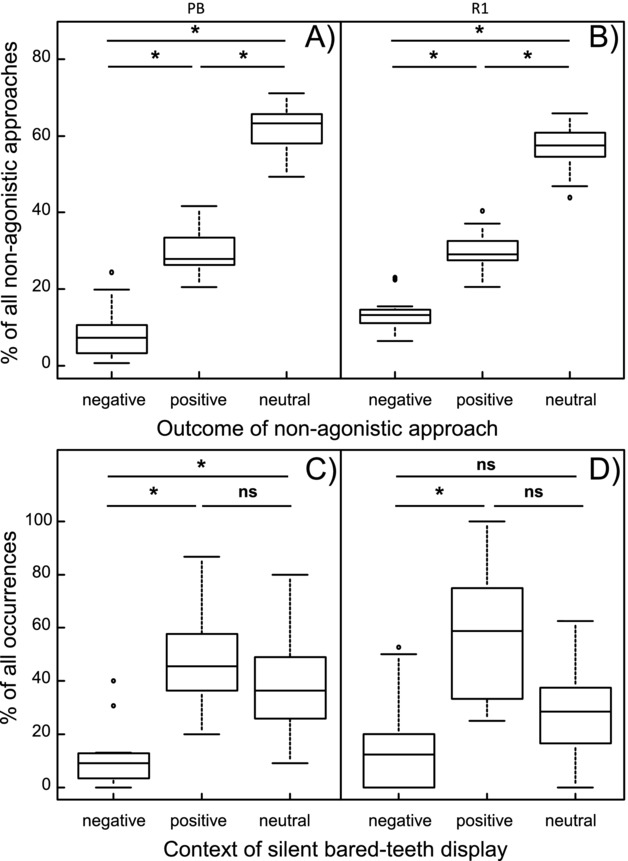
Two of the behavioral parameters indicators of social tolerance in macaques: outcome of approach (A and B) as a proportion of nonagonistic approaches and context of occurrence of silent bared-teeth display (C and D) as a proportion of all occurrences in PB (A and C panels; *N* = 15) and R1 (B and D panels; *N* = 21) groups (median, interquartiles and 1.5 interquartile range; post-hoc tests after Friedman, *observed difference > critical difference, ns = observed difference < critical difference).

Females in R1 group approached each other significantly more frequently than females in PB group (Mann–Whitney test: *N*_PB_ = 15, *N*_R1_ = 21, *U* = 245, *P* = 0.005; Table I). Nonagonistic approaches significantly led to different outcomes (Friedman chi-square test: PB: χ^2^ = 30, df = 2, *N* = 15, *P* < 0.001; R1: χ^2^ = 42, df = 2, *N* = 21, *P* < 0.001): most approaches did not result in any observable response (Fig. 2A, B; post-hoc tests: PB: *N* = 15, neutral/negative: observed difference = 30, critical difference = 13, neutral/positive: observed difference = 15, critical difference = 13; R1: *N* = 21, neutral/negative: observed difference = 42, critical difference = 16, neutral/positive: observed difference = 21, critical difference = 16). In addition, when females reacted to a close proximity approach, it was significantly more often positively than negatively (Fig. 2A, B; post-hoc tests: PB: *N* = 15, observed difference = 15, critical difference = 13; R1: *N* = 21, observed difference = 21, critical difference = 16; Table I).

Grooming and approach diversity indices were both close to 1 in both groups (Table I), indicating that females distributed their grooming bouts and approaches evenly across all female partners.

### Conciliatory Tendency

In total, we examined 285 PC–MC pairs (PB: 127, median per female = 8, range: 4–15; R1: 158, median per female = 7, range: 3–14). The median CCT with all affiliation (contact and noncontact) was 41% (median, range: 13–75%) in PB (4% dispersed, 45% attracted, 52% neutral) and 47% (median, range = 0–100%) in R1 (4% dispersed, 51% attracted, 46% neutral; see Table III for mean contact CCT).

### Dominance Hierarchy

Hierarchies in both groups were significantly linear (PB: h′ = 0.54 – 0.94, R1: h′ = 0.43 – 0.74, depending on the type of interactions, all *P*s < 0.01). In both groups and with all three types of agonistic interaction, all hierarchies were moderately but significantly steep (Table II, Appendix Table 9). DCIs were high for winner–loser and displacement interactions, indicating a high directionality of those dominance-related interactions (Table II). With all initiated aggressive acts, hierarchies were shallower compared to displacement interactions but rather similar to winner–loser interactions (Table II). DCIs were substantially lower however (Table II), indicating that, within a dyad, aggressive interactions could often be initiated by both members. Rank orders obtained with displacements and winner–loser interactions were similar (15 of 21 ranks in R1 and 10 of 15 ranks in PB). In contrast, with all initiated aggressions, only 3 of 21 ranks in R1 and 3 to 4 of 15 ranks in PB matched those established with decided interactions. Given that the displacement interaction matrices had the least number of unknown dyads, there were considered most reliable to establish rank orders.

**Table II:** Parameters of Hierarchies Considering Two Types of Dominance-Related Interactions

Group	Interaction types	*N* interactions	Percentage of unknown	Percentage of two-ways	Steepness *D*_ij_/P_ij_	DCI
PB (*N* = 15)	*Winner–loser*	207	23	2	0.420/0.631	98
	*Displacement*	561	4	18	0.693/0.895	89
	*All initiated*	360	10	42	0.398/0.576	62
R1 (*N* = 21)	*Winner–loser*	283	36	3	0.281/0.455	94
	*Displacement*	732	11	11	0.563/0.785	93
	*All initiated*	639	12	35	0.333/0.483	64

*Winner–Loser* interactions and *displacement* *i*nteractions, as well as *All Initiated* aggressive acts (see Text for definitions). Number of interactions recorded (*N* interactions), percentage of unknown relationships (Percentage Unknown) and of Two-Way Relationships in the matrices (Percentage Two-Ways; see Text for definition), Steepness Values with *D*_ij_ and *P*_ij_ indices (all significant) and Directional Consistency Indices (DCIs) are also given.

### Silent Bared-Teeth Display

Females in PB group showed silent bared-teeth displays significantly more frequently than females in R1 group (Mann–Whitney test: *U* = 228, *N*_PB_ = 15, *N*_R1_ = 21, *P* = 0.022; Table I). In both groups, females did not display this facial expression equally across interaction contexts (Friedman chi-square test: PB: χ^2^ = 13, df = 2, *N* = 15, *P* = 0.001; R1: χ^2^ = 19, df = 2, *N* = 21, *P* < 0.001). In PB group, females expressed silent bared-teeth least often in the negative context (post-hoc test: *N* = 15, neutral/negative: observed difference = 15, critical difference = 13, positive/negative: observed difference = 18, critical difference = 13). In R1 group, however, displays occurred similarly often in negative or positive contexts as compared to neutral contexts (post-hoc tests: *N* = 21, neutral/negative: observed difference = 13, critical difference = 16; neutral/positive: observed difference = 14, critical difference = 16), but occurred more often in a positive context than a negative one (post-hoc tests: positive/negative: observed difference = 28, critical difference = 16; Table I).

Diversity indices for the bared-teeth display were rather low (Table I), indicating that females showed this display to a specific set of female partners. Up/down indices for this behavior were not significantly different from 0.5 (value indicating no bias; Wilcoxon one-sample test: PB: *t* = 49, *N* = 15, *P* = 0.482; R1: *t* = 84, *N* = 21, *P* = 0.653), showing that females did not direct displays selectively up or down the hierarchy.

### Comparison within the Macaque Genus

Overall, most of the variables we measured fell within the range of data reported for captive or provisioned grade 4 female macaques (Table III).

**Table III:** Summary of Social Variables within Grade 4 Social Style

Variables	*M. nigra* (wild)^a^	*M. nigra* (captive)^b^	*M. tonkeana* (captive)^c^	*M. maurus* (captive/provisioned)^d^
Kin/nonkin	All	Nonkin	Nonkin	All
Bite (%)	2–3	8.9	0	2.6
Contact attack (%)	8–11	51.5	11.5	33.3
Counter aggression (%)	26–28	50.8–56.1	59.6	0
CCT (contact; %)	27–29	22.2	47.9	42.3
Steepness	0.281–0.693 (all)	0.257–0.817 (all)	0.332–0.653 (all)	0.397–0.545 (all)

References:

a this study.

b Petit et al. [1997], Petit [unpublished data], Micheletta [unpublished data], Balasubramaniam et al. [2012a].

c Thierry [1985]; Desportes and Thierry [unpublished data], Demaria and Thierry [2001].

d Matsumura [1996, 1998], Petit and Thierry [1992].

Mean percentages of Bite, Contact Attack, Counter Aggression, Mean CCT with Contact Affiliation and Steepness (with *D*_ij_ Indices) in females of different Grade 4 Macaque species: captive *M. nigra*, captive *M. tonkeana*, and captive and provisioned *M. maurus*, as compared to the studied Crested Macaques (*M. nigra*, Wild). Whether data have been published for Nonrelated Individuals Only (Nonkin) or All Individuals (All) is indicated in the first line. Steepness was computed regardless of kin relationships (All).

**Fig. 3:**
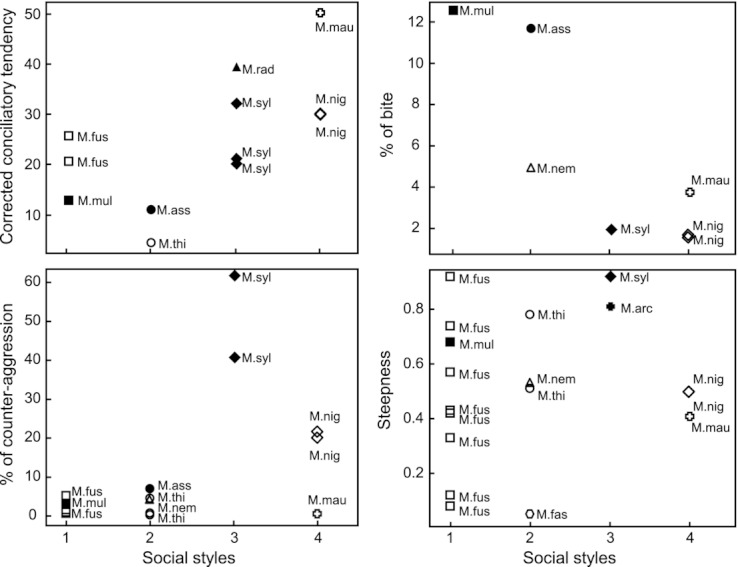
Variation of four social parameters according to social style grades, summarized across studies conducted on females under natural conditions. Means of CCT (upper left), percentage of agonistic interactions involving bites (upper right), or counter aggression (lower left) and steepness values (lower right) are represented. The four variables were extracted from published studies or calculated from (un)published data. The data set includes only studies of adult females, followed as focal individuals, under natural conditions, with or without provisioning. Data points within one grade represent means in different studies and/or different groups of the same or different species, abbreviated next to the data point. Different species have different symbols. Several data points may overlap when means are similar. (References: *M. fuscata* (M.fus, empty square): [Furuichi, 1983; Hanya et al., 2008; Hill & Okayasu, 1995; Koyama 2003; Kutsukake, 2000; Majolo et al., 2009; Mori et al., 1989; Nakamichi, 2003; Nakamichi & Shizawa, 2003; Oi 1988; Schino & Aureli, 2008); *M. mulatta* (M.mul, full square): [Cooper & Bernstein, 2008; Sade, 1972]; *M. assamensis* (M. ass, full circle): [Cooper & Bernstein, 2008]; *M. fascicularis* (M.fas, empty hexagon): [Gumert, 2000]; *M. thibetana* (M.thi, empty circle): [Berman et al., 2004; Berman et al., 2008]; *M. arctoides* (M.arc, full cross): [Estrada et al., 1977]; *M. nemestrina* (M.nem, empty triangle): [Oi, 1990]; *M. radiata* (M.rad, full triangle): [Cooper et al., 2007]; *M. sylvanus* (M.syl, full diamond): [Fa, 1985; Kuester & Paul, 1996; Patzelt et al., 2009; Thierry & Aureli, 2006; Thierry et al., 2008]; *M. maurus* (M.mau, empty cross): [Matsumura, 1996; Matsumura, 1998]; *M. nigra* (M.nig, empty diamond): this study, PB and R1 groups separately.)

The behavioral patterns we observed also fell within the range of data from other tolerant female macaques (grades 3 and 4, Fig. 3), and generally outside the range reported for more despotic ones (grades 1 and 2, Fig. 3). In contrast to females in more despotic species, aggressive interactions among the study females were of notably low intensity, frequently bidirectional, and often reconciled.

There is variation within grade and species, however. Compared to other populations of grade 4 macaques, the percentage of counter aggression and the CCT with contact affiliation in the study groups were low. Compared to some groups belonging to grade 3 (two groups of *M. sylvanus*, one of *M. radiata*), the study subjects also showed seemingly less counter aggression and fewer reconciled conflicts. Lastly, steepness values, which were expected to be lower in tolerant species compared to more despotic ones, varied too greatly within grade and even within species to show any clear relationship with the social style graded scale (Fig. 3).

## DISCUSSION

This study is the first comprehensive analysis of social behaviors of female crested macaques under natural conditions. We studied two wild groups and analyzed an extensive body of behavioral data on female social behavior; some of the variables were previously undocumented for grade 4 macaques. By focusing on the less studied tolerant end of the macaque social style spectrum, this study contributes to a better understanding of macaque societies.

Behavioral patterns observed in wild female crested macaques generally fit the definition of a tolerant social style: aggressive interactions are of low intensity, often bidirectional, and reconciled. The consistency of the observed patterns found in both study groups indicates the robustness of the results. We also found that much aggression was ignored or appeased and that affiliative interactions and approaches were frequent and evenly distributed among female partners. Power asymmetries between females were moderate. Displacement interactions were as frequent as aggressive interactions, seemed to be most reliable for computing hierarchy parameters and constitute a valid substitute to decided aggressive interactions to build hierarchies. Thus, social power appeared to be reinforced more commonly through weak rather than severe agonism. Since the occurrence of the silent bared-teeth display was linked neither to agonistic context nor to dominance rank, this facial expression did not constitute a signal of submission.

The degree of social tolerance in a society is best appreciated in comparison with other societies. Behavioral patterns of wild female crested macaques were very similar to those of captive conspecifics in particular, and of other grade 4 species in general, and substantially different from species in other grades. In addition, wild female crested macaques exchanged approaches and grooming evenly among a large network of female social partners, suggesting a low clustering in affiliation. This result is consistent with the work of Sueur and collaborators [2011], showing differences of affiliation network size and composition between tolerant and despotic social styles. This result would also be consistent with the usually less pronounced kin bias in affiliation characterizing other grade 4 macaques [Thierry et al., 1994; Thierry et al., 1990]. Three variables, namely, approach and grooming distribution and proportion of negative reaction upon approach, revealed a dimension of social tolerance that has never been quantified in macaques from grade 4 (for other grades see [Castles et al., 1996; Cooper & Bernstein, 2008; de Waal & Luttrell, 1989]). In comparison with female rhesus (grade 1) and Assamese (*M. assamensis*: grade 2) macaques, mean values of grooming diversity and percentage of negative reaction upon approach appear, respectively, higher and lower in the crested macaque females studied here [Cooper & Bernstein, 2008]. These measures of social tolerance thus seem to vary according to the species’ social style grade, and could provide reliable tools in the assessment of the degree of social tolerance.

Consistent with a high degree of social tolerance, female crested macaques expressed moderate power asymmetries. In this study, power asymmetries were more or less pronounced dependent on the type of interactions. Initiated aggressive acts yielded the same steepness values as winner–loser interactions but with a much lower DCI, showing that power asymmetries among females in those groups were not entirely due to capabilities of winning contests. Also, displacement interactions, more frequent and unidirectional than the two other types of interactions, pictured stronger asymmetries than decided aggressive interactions, indicating that power may be better asserted with low-intensity display than direct aggression. Similarly, Thierry et al. [1994] found that different agonistic variables yielded different hierarchical orders in captive Tonkean macaques. Such inconsistencies, evidenced both in captive and wild populations of grade 4 macaques and independent of observational effort, highlight the difficulty of reliably assessing hierarchical variables when a large proportion of aggressive interactions are represented by interactions with undecided outcome. Yet, those interactions may bear essential information about the dynamics of dyadic dominance relationships, perhaps representing negotiation interactions instead of or in addition to dominance interactions. Low-to-moderate power asymmetries, usually associated with an absence of formal submissive signals [Preuschoft & van Schaik, 2000], a pattern also found in this study, leave room for the negotiation of conflicts. Social negotiation may occur through the exchange of aggressive and affiliative signals within the same interaction, as we observed, or through the balance of aggressive and affiliative components in dominance relationships [de Waal, 1986]. These results suggest that it could be important to take these inconsistencies into account when analyzing further dyadic dominance relationships and how females deal with conflicts of interest.

The comparisons we carried out also highlighted intraspecies and intragrade variation. For example, counter aggression seemed to occur twice as frequently in captive populations of grade 4 macaques as compared to wild crested macaques, and was apparently absent in a provisioned group of moor macaques. These differences could reflect species differences or variation in demographic structure and/or living conditions. First, comparative studies showed that variation within species or grade is less pronounced than between species or grades, but species differences do exist [Balasubramaniam et al., 2012b; Thierry et al., 2008]. Second, even though the percentage of dyads without observed agonistic interactions was similar in all groups, groups of wild crested macaques were up to three times larger than groups of captive and provisioned populations, which could have resulted in different interaction dynamics. Moreover, in contrast to the other studies, our analyses were carried out disregarding kinship, information currently not available. Although the influence of kinship on social interactions appears relatively weak in grade 4 species [Demaria & Thierry, 2001; Matsumura & Okamoto, 1997], it may still be that the number of related individuals, and thus of potential allies, influenced the outcome of social interactions. Lastly, it has been shown that captivity or provisioning influence the rates, distribution, and intensity of contests through alteration of space available and/or food distribution [Asquith, 1989; Judge, 2000; Southwick et al., 1976; Wrangham, 1974]. Individuals subject to different degrees of competition would need to adapt their competition and conflict management strategies accordingly, albeit within their reaction norm. The extended choice of options of wild crested macaque females when responding to aggression (avoiding, ignoring, and affiliating) may constitute alternative tactics to retaliation and may better balance conflicts of interest. These results show that detailed analyses of responses to aggression can also help to reach a finer understanding of conflict management strategies, which have been shown to be tightly linked to social styles [Thierry et al., 2008].

Our comparative perspective is only descriptive and would need to incorporate formal phylogenetic analyses to be complete. We aimed here at scaling our data to the observed behavioral variation within the macaque genus, and not at testing differences between grades or species. Our conclusion is nevertheless consistent with other comparative studies, almost all controlling for phylogeny [de Waal, 1989; Thierry, 2000; Thierry et al., 2008; Thierry et al., 2000b]. Those studies showed, as is also illustrated in Figure 3, that the percentage of bites decreases with the social style grade whereas the percentage of counter aggression and the conciliatory tendency increase. However, high intraspecific and intragrade variation in steepness values, even among our study groups, prevents us from drawing any clear conclusion regarding their distributions along the gradient of social styles. Power asymmetries were expected to decrease from despotic to more tolerant species [Flack & de Waal, 2004; Thierry et al., 2008; van Schaik, 1989], a relationship confirmed in recent studies [Balasubramaniam et al., 2012b; Richter et al., 2009] using the steepness index developed by de Vries et al., [2006]. Our contradictory observation may indicate limitations of the steepness index when used to compare groups or species (e.g., influence of the proportion of unknown relationships in the matrix on the steepness value [de Vries et al., unpublished data; Klass & Cords, 2011]). Alternatively, the expected pattern may be revealed if data were controlled for phylogeny [Balasubramaniam et al., 2012a]. Our contradictory observation may also reflect the inclusion of different kind of agonistic interactions, for example only unidirectional (i.e., without counter aggression) or uni- and bidirectional [Balasubramaniam et al., 2012a].

Our comparative perspective also identified intraspecies variability, an issue that has puzzled animal behavior researchers for decades [Lott, 1991]. Our study groups, for example, also differed in their approach and silent bared-teeth rates, which may reveal different social dynamics, for example, differences in group size or group cohesion. This intraspecific variation is particularly well illustrated in the Japanese macaques, more variable in the degree of their interactions’ intensity and symmetry than expected [Nakagawa, 2010]. It is also now well appreciated that whereas the differences between the extreme ends of the social style gradient (grades 1 and 4) are clear-cut, the boundaries between and within the middle grades (2 and 3) are less distinct [Balasubramaniam et al., 2012b; Thierry, 2007].

Our study illustrates how consistent interrelated behavioral patterns are despite variation in environmental conditions. The social style concept thus seems to be valid and robust. The social style of wild female crested macaques now needs further investigation at the levels of social relationships and networks. We presently know that females exhibit a high degree of tolerance toward female conspecifics, and that they seem to form large social networks. This suggests that females presumably have more freedom to interact with social partners of their choice, regardless of dominance and kinship. However, dominance and kinship are two preponderant components of macaque societies, and the extent to which they influence these tolerant relationships in the wild is still unknown. It is also not known how tolerant females balance their levels of competition and cooperation in regard to ecological and demographic changes. Even though social styles and environmental conditions appear uncorrelated [Ménard, 2004], demographic and ecological factors may still participate in shaping the intensity, symmetry, distribution, and timing of social relationships between females in ways that are presently ill-understood [Henzi & Barrett, 2007; Henzi et al., 2009]. In macaque societies, the phylogenetic signal appears to be strong [Balasubramaniam et al., 2012a; Thierry et al., 2008; Thierry et al., 2000b], which suggests that internal constraints and phylogenetic history play an important role in their evolution. Nevertheless, investigating the different factors mentioned above as potential selection pressures on the evolution of different social styles is an important next step. Finally, the concept of social tolerance/despotism may be a feature that is not restricted to macaque societies but may be extended to other primate genera (e.g., [Hare et al., 2012; Leca et al., 2002]). This concept could help to unravel competitive and cooperative trade-offs faced by group members, shedding light onto the evolution of primate societies more generally.

## Appendix

**Table AI:** Matrix of Displacement Interactions in PB Group (*N* = 15—Row = Giver, Column = Receiver)

g/r	ap	bp	cp	dp	ep	fp	gp	hp	ip	jp	lp	np	rp	sp	yp
ap	0	0	1	0	0	1	0	0	0	0	0	1	3	0	3
bp	6	0	8	7	5	4	0	2	0	10	3	3	1	4	1
cp	2	0	0	4	1	2	0	0	0	0	0	3	6	0	4
dp	0	0	0	0	0	0	0	0	1	0	0	8	2	0	4
ep	4	1	1	4	0	1	0	0	0	1	0	5	4	0	0
fp	4	0	5	8	2	0	0	0	0	0	0	8	4	0	2
gp	2	1	1	1	4	0	0	1	4	4	0	2	2	3	1
hp	5	2	4	5	4	3	0	0	8	6	2	7	6	8	7
ip	2	10	0	5	10	9	0	2	0	12	0	8	5	15	3
jp	7	1	6	8	9	5	1	0	0	0	2	4	5	1	3
lp	3	10	9	7	2	6	3	7	11	7	0	17	5	20	5
np	1	0	0	0	0	0	0	0	0	0	0	0	4	0	5
rp	0	0	0	0	0	0	0	0	0	0	0	0	0	0	6
sp	3	6	8	6	7	5	0	0	0	5	3	4	2	0	4
yp	0	0	0	0	0	0	0	0	0	0	0	0	0	0	0

**Table AII:** Matrix of Winner–Loser Interactions in PB group (*N* = 15—Row = Giver, Column = Receiver)

g/r	ap	bp	cp	dp	ep	fp	gp	hp	ip	jp	lp	np	rp	sp	yp
ap	0	0	0	1	0	0	0	0	1	0	0	1	1	0	1
bp	3	0	1	0	0	1	0	0	0	3	0	2	0	0	0
cp	0	0	0	1	0	0	0	0	0	0	0	0	0	0	1
dp	0	0	0	0	0	0	0	0	0	0	0	3	2	0	1
ep	1	0	1	2	0	0	0	0	0	0	0	0	1	0	1
fp	1	0	0	1	0	0	0	0	0	0	0	3	2	0	0
gp	0	0	3	3	3	3	0	1	3	3	0	0	0	0	1
hp	1	2	4	2	4	0	0	0	2	3	0	2	2	4	3
ip	2	1	2	4	0	0	0	0	0	3	0	5	0	5	3
jp	1	0	3	1	3	1	0	0	0	0	0	1	2	0	3
lp	1	3	1	3	0	5	2	3	12	6	0	2	3	6	0
np	0	0	0	0	0	0	0	0	0	0	0	0	3	0	3
rp	0	0	0	0	0	0	0	0	0	0	0	0	0	0	7
sp	0	5	1	1	1	3	0	0	0	5	1	4	0	0	1
yp	0	0	0	0	0	0	0	0	0	0	0	0	0	0	0

**Table AIII:** Matrix of all Initiated Aggressions in PB Group (*N* = 15—Row = Giver, Column = Receiver)

g/r	ap	bp	cp	dp	ep	fp	gp	hp	ip	jp	lp	np	rp	sp	yp
ap	0	0	0	1	2	2	0	1	0	0	0	1	3	1	3
bp	3	0	2	0	0	1	0	3	4	2	2	2	0	0	0
cp	0	1	0	1	0	1	0	2	0	0	0	0	0	1	3
dp	0	2	4	0	1	0	0	3	2	2	2	4	2	2	1
ep	1	2	2	3	0	0	2	5	7	1	10	0	1	1	1
fp	1	2	1	3	0	0	0	1	1	0	1	6	2	0	1
gp	0	1	3	4	4	2	0	1	3	8	0	0	2	0	1
hp	1	1	4	2	4	0	0	0	4	6	0	2	2	6	2
ip	5	2	2	3	1	2	0	1	0	2	1	5	0	6	3
jp	1	7	3	2	5	1	1	1	12	0	6	3	2	4	4
lp	1	5	2	3	1	6	2	5	13	7	0	2	4	7	0
np	0	1	1	0	0	0	0	2	2	0	0	0	7	1	4
rp	0	0	0	0	0	0	0	0	0	0	0	0	0	1	8
sp	2	6	4	5	1	5	1	4	7	7	3	4	1	0	1
yp	0	0	0	0	0	0	0	1	0	0	0	0	0	0	0

**Table AIV:** Matrix of Displacement Interactions in R1 Group (*N* = 21–Row = Giver, Column = Receiver)

g/r	as	bs	cs	ds	es	gs	hs	Is	js	ks	ms	ns	os	ps	qs	rs	ss	ts	us	xs	ys
as	0	0	0	1	3	0	0	0	0	0	0	0	0	0	0	0	0	0	0	0	0
bs	5	0	0	2	3	1	0	0	1	3	0	0	0	7	0	0	2	0	3	0	7
cs	1	5	0	4	0	2	1	7	0	2	22	0	1	1	2	0	0	2	4	2	3
ds	0	0	0	0	1	0	0	0	0	0	0	0	0	0	0	0	0	1	0	1	0
es	0	0	0	4	0	0	0	0	0	0	0	0	0	0	0	0	1	0	1	0	1
gs	1	3	0	3	1	0	2	0	0	3	0	0	0	4	0	0	0	0	3	0	2
hs	2	5	0	2	3	5	0	0	3	2	4	0	0	1	0	0	1	1	4	1	5
is	1	1	1	3	2	0	3	0	1	3	5	0	0	1	0	0	0	0	4	5	3
js	2	2	0	4	2	4	0	0	0	2	0	0	0	3	0	0	1	0	3	0	3
ks	3	0	0	5	4	0	0	0	0	0	0	0	0	6	0	0	0	1	2	1	0
ms	1	5	1	5	3	1	0	0	5	3	0	0	0	3	0	0	0	7	2	1	2
ns	5	7	17	11	0	13	17	11	7	6	32	0	3	2	13	9	2	9	9	5	10
os	0	2	4	2	3	1	2	3	2	2	2	0	0	5	2	0	0	4	0	1	1
ps	4	0	1	5	3	0	0	0	0	0	0	0	0	0	0	0	3	0	0	0	0
qs	3	9	4	8	0	4	4	4	4	1	13	0	0	1	0	0	3	5	7	4	7
rs	1	1	7	3	0	1	1	0	1	0	2	0	1	2	2	0	0	1	5	1	1
ss	0	1	0	0	1	0	0	0	0	0	0	0	0	0	0	0	0	0	0	0	1
ts	2	1	1	5	1	0	0	0	3	3	0	0	0	0	0	0	1	0	4	0	2
us	5	0	0	5	1	0	0	0	0	4	0	0	0	3	0	0	0	0	0	0	5
xs	2	2	0	2	1	2	1	0	1	3	0	0	0	1	0	0	3	1	7	0	6
ys	1	1	0	2	3	0	0	0	1	0	1	0	0	2	0	0	1	0	0	0	0

**Table AV:** Matrix of Winner–Loser Interactions in R1 Group (*N* = 21—Row = Giver, Column = Receiver)

g/r	as	bs	cs	ds	es	gs	hs	is	js	ks	ms	ns	os	ps	qs	rs	ss	ts	us	xs	ys
as	0	0	0	2	3	0	1	0	1	0	0	0	0	0	0	0	0	0	0	0	0
bs	2	0	0	1	0	0	0	0	0	0	0	0	0	4	0	0	0	0	0	0	1
cs	1	0	0	0	0	1	1	1	0	0	1	0	0	0	2	1	0	2	1	0	1
ds	0	0	0	0	0	0	0	0	0	0	0	0	0	0	0	0	0	0	0	0	0
es	0	0	0	0	0	0	0	0	0	1	0	0	0	0	0	0	1	0	0	0	0
gs	0	0	0	0	0	0	0	0	0	4	0	0	0	0	0	0	0	0	5	0	3
hs	1	1	0	5	1	2	0	0	0	0	9	0	0	1	0	0	1	2	3	2	2
is	1	0	0	0	1	0	1	0	0	0	0	0	0	1	0	0	0	0	0	3	0
js	5	0	0	0	3	3	0	0	0	1	0	0	0	1	0	0	0	0	1	0	0
ks	2	0	0	4	1	0	0	0	0	0	0	0	0	2	0	0	1	0	0	0	1
ms	5	0	0	5	0	0	2	0	0	2	0	0	0	1	0	0	1	4	2	2	0
ns	2	0	2	0	0	0	1	0	1	4	0	0	2	1	3	2	0	4	1	1	1
os	1	2	2	2	0	2	3	4	0	1	2	0	0	2	0	0	0	2	2	2	1
ps	1	0	0	2	0	0	0	0	0	0	0	0	0	0	0	0	4	0	0	0	0
qs	9	1	0	4	0	1	2	3	0	4	2	0	0	2	0	0	0	1	2	0	2
rs	2	3	0	2	0	0	1	1	3	0	1	0	0	0	1	0	1	1	0	0	0
ss	2	0	0	0	1	0	0	0	0	0	0	0	0	0	0	0	0	0	0	0	0
ts	3	0	0	1	1	0	0	0	1	2	0	0	1	2	0	0	1	0	0	0	0
us	2	0	0	5	0	0	1	0	0	4	0	0	0	3	0	0	1	0	0	0	3
xs	1	0	0	0	0	1	0	0	1	1	0	0	0	1	0	0	0	0	1	0	1
ys	2	0	0	1	1	0	0	0	0	0	0	0	0	1	0	0	1	0	0	0	0

**Table AVI:** Matrix of all Initiated Aggressions in R1 Group (*N* = 21—Row = Giver, Column = Receiver)

g/r	as	bs	cs	ds	es	gs	hs	is	js	ks	ms	ns	os	ps	qs	rs	ss	ts	us	xs	ys
as	0	0	1	2	4	0	2	2	0	0	1	0	0	0	2	0	0	0	0	0	0
bs	6	0	0	1	3	0	0	0	0	2	0	0	0	5	0	0	0	1	1	0	3
cs	3	1	0	1	0	2	3	1	1	0	7	2	0	1	1	1	0	2	1	0	1
ds	0	0	2	0	1	1	4	2	0	0	7	0	0	1	1	0	0	1	0	1	0
es	0	0	0	0	0	0	1	0	0	1	0	0	1	1	1	0	1	0	0	0	1
gs	1	0	1	0	1	0	2	1	0	8	0	0	0	3	0	0	0	0	7	2	2
hs	4	3	7	4	0	3	0	2	1	1	9	7	1	1	5	1	1	1	2	1	2
is	2	0	0	0	1	1	2	0	3	0	0	2	0	2	0	3	0	1	0	5	0
js	6	0	13	2	4	2	8	8	0	5	14	5	4	1	9	1	0	1	1	0	1
ks	4	0	6	6	3	2	0	0	0	0	3	1	1	2	3	0	1	2	2	0	2
ms	18	0	20	7	1	0	7	0	4	3	0	4	2	2	3	1	1	8	6	2	0
ns	2	1	3	2	0	0	0	1	2	4	3	0	3	2	3	3	0	4	3	3	1
os	1	2	3	1	1	2	5	4	0	1	4	0	0	2	4	0	0	2	3	3	1
ps	2	0	1	3	1	0	0	0	0	0	3	0	0	0	0	0	4	0	0	0	0
qs	10	1	1	5	1	1	2	3	1	3	3	1	1	2	0	0	1	3	2	1	3
rs	2	2	1	1	1	0	4	0	3	2	3	1	1	0	1	0	2	1	0	0	1
ss	1	0	0	3	1	0	0	0	0	0	0	0	0	0	0	1	0	0	1	0	0
ts	4	0	0	1	0	0	1	0	2	1	0	0	2	5	0	1	1	0	0	0	1
us	6	0	4	9	3	0	8	1	2	4	5	0	0	3	1	1	1	0	0	1	2
xs	3	0	0	2	4	2	0	0	2	3	0	0	0	3	0	0	1	0	2	0	2
ys	4	0	1	5	2	0	1	3	0	0	12	1	0	3	1	0	1	2	0	0	0
